# Navigating Dynamic Power Relations in Patient Involvement: Researchers' Perspectives From University Research

**DOI:** 10.1111/hex.70600

**Published:** 2026-02-11

**Authors:** Eeva Aromaa, Päivi Eriksson, Tero Montonen, Pasi Hirvonen

**Affiliations:** ^1^ Business School University of Eastern Finland Kuopio Finland; ^2^ Department of Social Sciences University of Eastern Finland Kuopio Finland

**Keywords:** dynamic, patient involvement, power, qualitative research, relational, research, tension

## Abstract

**Background:**

Addressing power issues in research involvement is needed for advancing equitable relationships between patients and researchers. While previous studies acknowledge power challenges in patient involvement, few approach power as a dynamic phenomenon in which multiple and shifting power relations coexist.

**Objective:**

To examine how researchers navigate three power relations identified in patient involvement research – power with, power to, and power over – and associated tensions.

**Methods:**

Sixteen purposively sampled life science, social science and clinical researchers from two Finnish universities participated in thematic interviews. Interview transcripts were analysed thematically.

**Results:**

Researchers navigated power relations through three tensions in patient involvement: (1) tokenism versus co‐creation, (2) institutional structures versus everyday complexities, and (3) conflict versus reflexivity. In tokenism versus co‐creation, gatekeeping (power over) and openness to patient ideas (power to) co‐existed, but also evolved towards co‐researchership (power with). In institutional versus everyday complexities, mediated representation replaced direct involvement, reinforcing hierarchies; funders' recommendations encouraged collaboration but could also consolidate power over through procedural control. In conflict versus reflexivity, power over was evident in patients' rejection of assigned roles and interactional and relational challenges within involvement practices that reproduced hierarchical dynamics.

**Conclusions:**

The study contributes to the discussion of power challenges in patient involvement by illustrating how researchers simultaneously enact, reproduce and resist multiple power relations. A dynamic view of power clarifies researchers' roles in shaping patients' involvement in research.

**Patient or Public Contribution:**

The first author is a trained Expert by Experience patient‐researcher with extensive involvement in researcher–patient collaboration across health care and health research contexts. She has participated in all study phases and had a major role in data collection and analysis.

## 1 Introduction

Power challenges have been identified as significant barriers to both collaborative knowledge production between health researchers and lay contributors and the democratic participation of patients in research [1, 2, 3]. Although various co‐approaches have become more common in countries with a longer history of participatory health research [4, 5], collaboration often remains driven by researchers rather than patients [6]. As researchers define research agendas, their experiences and expectations of patient involvement play a key role in shaping how collaborative ideals are enacted in everyday research practice [7]. Consequently, researchers' understandings significantly shape the possibilities for building deeper and more sustained co‐approaches.

From the perspective of power in researcher–patient relationships, previous studies have documented persistent challenges in shifting hierarchical structures towards more equal and empowering modes of collaboration. Challenges include researchers' neglect of their paternalistic position of power and expertise and their tendency to maintain the status quo [8, 9], resistance to sharing control over the research process and engaging in knowledge co‐creation with patients [10], power imbalances rooted in wider social and cultural contexts [11], differences in research knowledge [12], and involvement practices that fall short of genuine empowerment [13]. However, much of this literature, particularly focusing on researchers' power over patients, has conceptualised power as a static rather than dynamic phenomenon. By a static view of power, we refer to approaches that treat power as a fixed attribute or position – mostly held by researchers and imposed on patients – rather than as something negotiated, situational, and relationally produced over time. Although important exceptions exist [14, 15], a gap remains in empirical research that examines the interplay of power relations in patient involvement.

In this study, we approach power as a relational and dynamic phenomenon that emerges through social practices and discursive processes, while also stabilising in organisational and institutional structures [16]. Simple or linear conceptualisations of power do not adequately capture its complexity, as power relations may shift over time, a perspective supported in conceptual work [17] and in empirical studies of other professional–lay contributor contexts [18]. Yet, empirical studies applying such a dynamic view of power in health research remain scarce [14, 15].

The purpose of this study is to explore researchers' experiences and expectations of patient involvement in the Finnish university research context, with a focus on how researchers navigate multiple power relations and related tensions. Examining the researcher perspective is important, as it helps to illuminate how hierarchies may be inadvertently reproduced even in contexts explicitly oriented towards collaboration. A more nuanced understanding of how power is navigated can inform the design of involvement practices that are contextually appropriate, transparent, and mutually beneficial. For patients, greater insight into how researchers interpret and manage power relations may contribute to clearer expectations and more meaningful opportunities for influence in shaping research agendas and outcomes.

The analysis draws on three power relations identified in prior research [19, 20, 21, 22]: *power over*, where researchers dominate or limit patients' influence, often through control over participation terms and the suppression of alternative ideas; *power to*, where researchers enable patients' contributions within bounded and often researcher‐defined opportunities; and *power with*, where patients and researchers share decision‐making through mutual collaboration, dialogue, and negotiation. Rather than addressing power expressions within sequential stages, we explore dynamic power relations as multiple, coexisting and intertwined with tensions. By tension, we refer to a persistent strain arising from the interplay of competing experiences, expectations with power relations. These tensions cannot be fully resolved but must be continually navigated [23].

We address the following research question: *How do researchers navigate power relations and associated tensions in the context of patient involvement in research?*

## 2 Methods

### 2.1 Setting and Participants

Drawing on a broader dataset on researchers' stakeholder relationships, this study focuses researchers' experiences of and expectations to patient and public involvement (PPI). This is the term used in the Finnish context for the involvement of patients as partners in health research, as they contribute to knowledge production and decision‐making beyond their role as research subjects [5]. We use the term patient, as it reflects how research involvement was understood in Finnish health research when we collected our data. It also reflects how involvement language is used in our data. We acknowledge, however, that the term patient is contested and that alternative terms can be preferred in other contexts (e.g., citizens, knowledge or service users).

The 16 researchers participating in our study were purposively sampled [24] based on their prior experience with and future interest in PPI. The participants were selected from two Finnish universities with a strategic commitment to medical and health research. The participants included life scientist (*n* = 6) from fields of biomedicine, biology and pharmacy; clinical researchers (*n* = 5) from medicine, and social scientists (*n* = 5) engaged in health research (Table 1). Their level of prior experience in PPI varied, but all had an interest and/or felt the pressure to involve patients in future research. None of the interviewees were identified in the interviews as a patient or health research service user.

**Table 1:** Demographics of participants interviewed in this study.

	Participants *(n* = 16)
Age, years	
Median age (range)	44 (31–64)
Gender	*n* (%)^a^
Female	11 (69%)
Male	5 (31%)
Position	
Professor (full, associate, assistant)	11 (65%)
Project researcher	6 (35%)
Scientific background	
Life science	6 (38%)
Clinical research	5 (31%)
Social science	5 (31%)
Duration of interviews	
Mean duration (range)	40 min (30–60 min)

^a^ All figures are presented as *n* (%) unless otherwise stated.

Most participants were academic scholars (assistant, associate or full professors), affording them autonomy in designing research projects and patient involvement in those. A smaller number were project researchers that often lack such academic independence. In the Finnish context, the title of assistant professor and associate professor refer to tenure‐track academic positions with key responsibilities in research and teaching. Most of the participants were women, which aligns with the findings that patient involvement is often in the interest of and on the responsibility of female scholars [25]. We collected basic demographic characteristics (age, gender, discipline, position) only, as our analytical focus was not on individual or group attributes.

### 2.2 Data Collection

Two authors contacted potential participants via email, outlining the purpose of the study and explaining the informed consent process, including confidentiality and data security measures. Interviews were conducted via Zoom between September 2021 and September 2023, lasting between 30 and 60 min, with median of 40 min. All interviews were audio‐recorded and transcribed verbatim.

Thematic and conversational interviews encouraged participants to share their experiences and expectations about stakeholder collaboration, including patient involvement, and to provide examples of patient involvement in their research whenever possible. The interviewees were not explicitly involved in formulating the research question guiding this study. Instead, interviews were intentionally open‐ended, allowing participants to foreground issues they considered salient. The research question focusing on power relations subsequently emerged through the authors' inductive analytical process grounded in the interview data.

### 2.3 Data Analysis

We followed reflexive thematic analysis as outlined by [26, 27], comprising six phases. First, all authors familiarised themselves with the transcribed data. Second, we focused on sections in which interviewees discussed their understandings and experiences of patient involvement in research. Initial coding of semantic units was conducted independently by all authors, using an inductive approach to capture the meanings conveyed by participants [24, 28]. Initial codes were compared and discussed among the authors to reach joint interpretation. These inductively generated codes were then abductively related to three power relations identified in prior research (e.g [22]), with a focus on their co‐occurrence. Codes and emerging interpretations were shared among the authors to reach analytical consensus. Both explicit and implicit understandings of power were considered.

Third, we developed initial themes by grouping the codes and evaluated their accuracy within the author team. Fourth, themes were refined to identify patterns of shared meaning, leading to an overarching structure of how researchers navigated power relations within three specific tensions characterising patient involvement (1) tokenism versus co‐creation, (2) institutional structures versus everyday complexities, and (3) conflict versus reflexivity. Fifth, analysis remained iterative, with ongoing discussions to refine interpretations of power, ensuring the consistency of the findings. Sixth, during the writing process, some data extracts were re‐examined and re‐analysed, reflecting the iterative nature of the process.

## 3 Findings

Researchers navigated between the three power relations (power over, power to and power with) through the following tensions: (1) tokenism versus co‐creation, (2) institutional structures versus everyday complexities, and (3) conflict versus reflexivity (Table 2). Although interviewees represented different disciplinary backgrounds and academic positions, we did not observe systematic differences in how power relations were navigated.

**Table 2:** Summary of tensions, power relations and results.

Tension	Power relations	Results
Tokenism versus co‐creation	Power over: Researchers define terms, boundaries, and scope of involvement Power to: Bounded opportunities for involvement granted by researchers Power with: Shared authority and co‐production in research	Tokenism may persist beneath the language of participation, with decision‐making authority retained by researchers. Patients can influence specific aspects of research (e.g., framing questions) but remain within researcher‐defined limits. Over time, relationships can shift towards genuine collaboration, for example, evolving from tokenistic presence to co‐authorship.
Institutional structures versus everyday complexities	Power over: Institutional expectations mediated through researcher authority Power to: Institutional encouragement for patient inclusion Power with: Continuous collaboration across project stages	Institutional pressures may reinforce researcher control by prescribing forms of involvement without enabling shared decision‐making. Funding bodies' recommendations or requirements may open opportunities for patient influence but within bounded frames. Structural pushes for ongoing involvement can promote shared authority, but practical constraints often limit realisation.
Conflict versus reflexivity	Power over: Researchers' perspectives shape participation Power to: Enabling patient voice through invitations Power with: Shared decision‐making with reflexivity	Patients may resist predefined roles, leading to reassertion of researcher authority. Open calls or invitations can increase influence, but active patient agency may challenge existing processes. Difficult to achieve in practice when assertiveness or organisational structures disrupt collaborative intentions.

*Source:* Authors' own.

### 3.1 Navigating Tokenism Versus Co‐Creation

Researchers described how patient involvement in research sat on a spectrum, from symbolic inclusion to substantive collaboration. However, analysis showed how power relations coexisted and shifted in their descriptions. Accordingly, scientific gatekeeping (power over) and openness to patient ideas (power to) manifested simultaneously, and tokenistic arrangements could transform into co‐authorship (power with). The following quote demonstrates a researcher's understanding on this continuum and the possibility that language of participation may conceal power over dynamics.

Patient involvement spans various levels. I think co‐research is at one end of the spectrum, while at the other end, you have something like consulting through hearings. The real question is whether we are genuinely involving patients or simply engaging in tokenism because we feel we ought to.

(Interviewee 3)

Here, co‐research is associated with power with, shared knowledge production, while consultative hearings suggest power to, where patients may advise but lack decision‐making power. The central question about genuineness reveals the risk that power over remains embedded even in participatory language. This continuum requires active navigation, with researchers assessing whether their practices reinforce or challenge hierarchies.

Some researchers also suggested that limited involvement could be contextually sufficient:

It [patient involvement] has been done forever, especially when it has been understood as simply asking patients whether a medicine tastes bad or not. Then, it's reported that patients have been included. I hate that term, been included! When people are included, it's imposed from above, and they aren't truly given the chance to participate. Sometimes, though, that approach is fine. Sometimes it's sufficient, and it's all that's needed.

(Interviewee 2)

This quote critiques superficial consultation as power over, where inclusion is imposed without influence. Yet the acknowledgement that such involvement can be sufficient in certain contexts points to conditional power, and to the coexistence of all three expressions of power within the same relationship. Long‐term arrangements, however, were described to shift toward deeper collaboration:

I have been involved in [international patient organisation's] projects for 15–20 years, and there has always been a patient included in those groups. One could question whether it was, in some ways, tokenism. At the start of a particular research project, while formulating research questions, a patient shared their personal story. Now, we are co‐authoring an article with them and an international research group. So, this collaboration has evolved throughout the project.

(Interviewee 3)

Here, tokenistic power over at the outset transitions to power to through patient input, and ultimately to power with, in the form of co‐authorship. The evolution demonstrates that power modes are not fixed but temporally fluid. Researchers further reflected critically on who is seen as a credible and desirable participant:

It is important to ensure that the patient involved is capable of processing and evaluating data with a scientifically objective mindset. However, is that really what we are looking for? Involving patients can also lead to surprises, brilliant new ideas that we [researchers] might not have thought of ourselves.

(Interviewee 16)

This statement reinforces power over by privileging scientific norms as the measure of legitimacy. The subsequent recognition of patients' creative contributions suggests power to, and openness to mutual learning signals the possibility of power with. These dynamics coexist, illustrating how epistemic boundaries are both maintained and questioned.

Finally, language use emerged as a site of negotiation:

Research groups often use terminology that laypeople don't understand. There must be a trusting atmosphere where patients feel comfortable saying, “I don't understand, could you explain?” This requires in‐person interactions, collaboration, open conversations, and ongoing reflection on expectations and roles.

(Interviewee 6)

In this quote, technical language reflects power over but creating a trusting atmosphere grants power to patients by enabling them to challenge linguistic barriers. The emphasis on collaboration and reflection points toward power with, showing how relational power can be layered in everyday practice.

### 3.2 Navigating Institutional Structures Versus Everyday Complexities

In principle, institutional requirements promoted power with, yet reinforced power over through procedural control. Researchers found it difficult to manage funder expectations, and direct patient involvement was sometimes replaced by mediated representation, reinforcing hierarchies. Thus, researchers found themselves negotiating between formal institutional expectations and the lived realities of patient involvement. While funders' recommendations encouraged more collaborative power relations, these same structures could reinforce power over through procedural control and standardisation.

One thing, of course, is the funding. Patient involvement is not necessarily required for funding, but it is at least recommended as a good practice. Some level of involvement, beyond the traditional sense, is encouraged. Integrating it into research plans seems to be the preferred approach.

(Interviewee 4)

This quote demonstrates a subtle interplay between power over and power to. Although external recommendations nudge researchers toward inclusion, the discretion over extent and form remains with the researcher, preserving hierarchical authority. The phrase “some level of involvement” reflects conditional empowerment, in which patients are valued contributors but only within researcher‐defined limits. Notably absent is power with, as there is no indication of shared decision‐making at the level of agenda‐setting or project governance. Formal requirements were described to lead to new forms of control:

Today, these EU projects… they want to include patients from the planning phase of the research project all the way through to its implementation and reporting. I would consider that quite a complex undertaking. In many ways, it's certainly a rather difficult thing to manage for both parties.

(Interviewee 8)

Here, funders' push for continuous involvement aligns with power with ideals. Yet, describing the process as “complex” and “difficult to manage” signals that researchers may need to retain control to ensure project feasibility, reasserting power over. The intention to give power to patients across all stages is tempered by practical constraints, showing how institutionalised participation can simultaneously expand and restrict influence.

However, in some cases, structural arrangements were seen to actively mediate patient access:

When I started assembling the group, a challenge was that many patient advocacy organisations sent only their representatives, despite my clear request for participants from the target group.

(Interviewee 1)

In this quote, the researcher's aim was to grant power to patients directly by involving those with lived experience. However, the intermediation of advocacy organisations reintroduced power over by controlling who could participate. Even well‐intentioned representation can reinforce hierarchies, as gatekeepers filter whose voices are heard. Attempts to establish power with through direct collaboration were obstructed, demonstrating the tension between procedural legitimacy and experiential authenticity.

### 3.3 Navigating Conflict Versus Reflexivity

Conflict versus reflexivity emerged when researchers experienced patients resisting the roles offered to them, or when everyday interactions complicated participatory ideals. Attempts to grant power to were sometimes resisted by patients asserting their own terms, unsettling researcher control and prompting researchers to reflect on the limits of their involvement practices. For instance, interactional difficulties and interpersonal tensions could lead to reassertion of power over, rather than opening space for reflexive renegotiation of roles. Practices labelled as “involvement” sometimes masked hierarchical control, revealing a tension between reflexive intentions and conflictual encounters.

Researchers also experienced situations where attempts to grant power to were challenged by patients asserting their own agendas. These moments forced researchers to choose between engaging reflexively with the contestation or re‐establishing procedural control to stabilise the research process.

Through a newspaper ad, individuals were invited to share their stories. Among them, a group of “justice fighters” stood out for their strong advocacy of their ideas. They contacted us repeatedly, sending various materials and requests to be included in the study. These highly active individuals can sometimes be challenging.

(Interviewee 3)

The open invitation reflects an effort to extend power to patients by offering them a platform. Yet, the researcher experienced that the “justice fighters” sought to define their own terms of involvement, challenging power over dynamics and destabilising the researcher's role as gatekeeper. This form of interaction, though aligned with participatory democracy, was interpreted by the researcher to test the boundaries of process control, triggering a conflict between maintaining authority and engaging in reflexive accommodation. In this context, power with became difficult to sustain without confrontation. Furthermore, interactional dynamics were reported to affect collaboration:

There have been discussions about the “nothing about us without us” philosophy. However, the actual group of patients can sometimes be aggressive and demanding, making it difficult to implement this ideal. The public perception of illness often differs greatly from reality.

(Interviewee 13)

Here, the slogan embodies power to and power with ideals, but the researcher's account suggests that interpersonal difficulties can lead to a reassertion of power over, rather than reflexive adjustment of involvement practices. By framing patients as aggressive or demanding, the researcher implicitly justifies retaining control, thereby resolving tension through authority rather than reflexivity. Overall, this tension highlights the gap between aspirational democratic rhetoric and the contours of day‐to‐day practice.

Finally, researchers acknowledged that power over could persist beneath the surface of formal involvement, even in situations framed as reflective or participatory:

Patient involvement can often be more about the researcher dictating, from their own perspective, what the study needs.

(Interviewee 5)

This reflection shows that even when labelled as involvement, processes can remain researcher‐led, with patient input limited to predefined areas. Such arrangements offer constrained power to, but power with is largely absent, demonstrating how unresolved conflict may foreclose reflexive engagement. This underlines the risk that institutionalised participation may reproduce, rather than dismantle, entrenched hierarchies.

## 4 Discussion

This study makes an original contribution to patient involvement in research by empirically demonstrating that power over, power to, and power with are not necessarily discrete or sequential phases but may coexist within the same situation. In other words, while early conceptualisations depict power shifts as a progression along a continuum from hierarchical control (power over) towards shared authority (power with), positioning power to as an intermediate step [22], our findings challenge such linear model. Rather than replacing one expression of power with another, multiple power relations may be simultaneously enacted, with effects that are contingent on situational aims, expectations, and constraints.

By approaching power as relational and dynamic [14, 17, 21], we offer nuanced insight of how researchers enact authority in patient involvement. Our findings suggest that researchers do not simply hold or relinquish power but recalibrate configurations of power relations in response to collaboration trajectories, institutional requirements, and perceived practicalities. In this sense, maintaining power over to protect research integrity, comply with regulatory demands, or ensure feasibility, even while fostering power to or power with. This finding challenges simplified empowerment narratives that equate patient involvement with the absence of hierarchy.

A key theoretical contribution of this study lies in analysing the interplay of three power relations across three tensions in patient involvement: tokenism versus co‐creation, institutional structures versus everyday complexities, and conflict versus reflexivity. This framing demonstrates that power relations rarely unfold as linear stages but instead may co‐occur within the same tension. It also highlights how power over may re‐emerge even in explicitly collaborative settings, underscoring the fragility of shared authority.

### 4.1 Relationship to Previous Research

Earlier studies of patient involvement have often taken a static approach to power, focusing on the persistence of researcher authority and other barriers to democratic collaboration [8, 9, 10]. While valuable, such accounts may obscure the simultaneous presence and interaction of multiple power relations. Our findings address this gap by illustrating how tokenistic inclusion (power over), bounded influence (power to), and emerging co‐researchership (power with) can coexist, each linked to specific moments and interpersonal dynamics.

Our analysis extends participatory research that conceptualises power as relational and dynamic [14, 17, 18]. This perspective helps explain why specific involvement practices may be maintained, adapted, and re‐initiated as collaboration evolves. The reassertion of power over, even after moves towards power with supports arguments that power is embedded in organisational and societal structures [29], making it resistant to complete transformation through individual efforts alone.

This study also adds nuance to debates on tokenism in patient involvement [2]. While confirming that tokenistic practices remain common [13], our findings show that tokenism may, in some cases, function as a provisional arrangement that evolves into more substantive collaboration. Such shift was triggered by relational moments that altered researchers' perceptions of patients' contributions, suggesting that tokenism and collaboration are not necessarily mutually exclusive but can be connected phases within an evolving researcher–patient relationship.

Institutional structures emerged as both enabling and constraining. Consistent with earlier research [30], our findings show that funder requirements can legitimise involvement while simultaneously reinforcing power over through standardisation, mediated representation, and administrative complexity. These conditions shape not only whether patients are involved, but also how involvement is organised and on whose terms.

The theme of conflict versus reflexivity adds an underexplored dimension to patient involvement scholarship. While resistance from patients has been noted previously (e.g. [31]), our findings show how assertive patient agency can disrupt involvement practices, creating tensions that researchers need to deal with. These moments may open space for power with, but they may also prompt researchers to reassert control to protect project feasibility.

Overall, participatory research is more complex than the ideals it is grounded in, and collaboration with diverse patient groups gives rise to tensions in practice. These tensions do not originate in patients or researchers as such but emerge within interactional contexts shaped by differing preferences, expectations, and modes of engagement, as well as by the communication practices, institutional constraints, and implicit expectations of both parties.

### 4.2 Contributions to Understanding Each Tension

In relation to tokenism and co‐creation, our findings challenge the assumption that power over must be eliminated for co‐creation to occur. In some contexts, maintaining limited hierarchical control was framed as necessary, particularly in technically or regulatorily constrained projects. Here, power to functioned as a legitimate and pragmatic mode of involvement, consistent with Avelino and Wittmayer's [17] observation that power can operate simultaneously as a resource and a constraint.

In navigating institutional structures and everyday complexities, our findings confirm that funder requirements can legitimise involvement while also prompting new expressions of power over [30, 32]. In these situations, power with ideals frequently coexisted with centralised project control, reflecting the practical demands of project management and accountability.

In navigating conflict versus reflexivity, the findings extend earlier work [8, 12] by showing that contestation may function as a driver of reflexivity rather than merely a barrier. However, realising this potential requires facilitation, negotiation, and boundary‐setting skills that are rarely foregrounded in patient involvement guidance, increasing the risk that conflict is resolved through authority rather than reflexive adjustment.

In a nutshell, this study advances understanding of patient involvement by reframing power not as a linear shift from control to equality, but as a dynamic and situationally contingent configuration of power relations that researchers need to navigate. Shared authority thus emerges as negotiated and potentially fragile, and subject to reconfiguration in response to both institutional and interpersonal pressures.

### 4.3 Practical Implications

The findings suggest that patient involvement can be approached as an evolving process in which multiple power relations are acknowledged. Recognising that power over, power to, and power with can coexist, and that their relative balance may shift over time, can help researchers and patients to develop shared expectations and reduce misunderstandings. This perspective foregrounds transparency regarding the scope and limits of participation, rather than assuming that collaboration automatically entails shared decision‐making.

Temporal flexibility should be built into involvement processes, allowing relationships to mature from symbolic inclusion towards substantive co‐creation as opportunities for deeper engagement emerge. At the same time, limited participation modes may be appropriate in certain research contexts, provided that these are agreed upon transparently and communicated clearly.

Institutions and funders play a critical role in shaping the structural conditions for involvement. While policy mandates and funding requirements can catalyse inclusion, they should be designed to support rather than constrain collaboration. Attention should be paid to minimising organisational gatekeeping that filters patient voices and limits direct interaction between researchers and patients. This includes reflecting on how representative structures and procedural requirements privilege certain perspectives while marginalising others.

Finally, contested and tensioned dynamics can be anticipated as part of everyday involvement practice. Rather than being viewed as evidence of failure, tensions can serve as opportunities for rethinking processes and roles. Equipping researchers with facilitation and conflict management skills is therefore essential for sustaining collaborative relationships over time. In a power with orientation, responsibility for addressing tensions does not rest solely with researchers but is shared with patients, thus requiring collective negotiation and reflexivity.

### 4.4 Strengths and Limitations

A strength of this study lies in its focus on researchers' experiences and expectations of patient involvement analysed through a theoretically informed framework of power. The purposive sample encompassed researchers from three disciplines within two universities, providing a rich and varied dataset. The thematic analysis supported both inductively emerged questions and theory‐driven interpretation. The study seeks analytical rather than statistical generalisability, aiming to provide transferable insights relevant to similar contexts.

The perspectives captured in this study are those of researchers rather than patients, meaning that the dynamics described represent one side of the relationship. Future studies should incorporate patient perspectives, which would provide a more holistic understanding of how power relations are negotiated between both parties [23]. Finally, attention to the interplay of power relations foregrounded relationality, but potentially limited attention to structural aspects of power, which remain important avenues for future research.

## 5 Conclusion

This study underscores how patient involvement can be understood as navigation of power relations, rather than as a set of activities that can be fixed through design or policy alone. Involvement frameworks that assume a straightforward move from researcher control to shared decision‐making risk overlooking how authority is shaped in everyday interactions, institutional requirements, and moments of disagreement. For those engaged in patient involvement, this perspective highlights the importance of reflexivity as a shared responsibility. Researchers, patients, and organisations need to recognise that tensions are not necessarily signs of failure, but often indicators of underlying power dynamics that require attention. Addressing these dynamics can help prevent involvement from reverting to symbolic participation when collaboration becomes challenging.

More broadly, a dynamic view of power invites more realistic and context‐sensitive expectations of patient involvement. Rather than equating meaningful involvement with the absence of hierarchy, it encourages attention to how participatory relationships are built, maintained, and renegotiated over time. Such an approach can support patient involvement that is not only procedurally compliant, but also relationally grounded and responsive to the realities of health research practice.

## Author Contributions

**Eeva Aromaa:** conceptualisation, data curation, formal analysis, investigation, methodology, project administration, validation, writing – original draft preparation, writing – review and editing. **Päivi Eriksson:** conceptualisation, formal analysis, funding acquisition, investigation, methodology, writing – original draft preparation, writing – review and editing. **Tero Montonen:** conceptualisation, formal analysis, investigation, methodology, writing – original draft preparation, writing – review and editing. **Pasi Hirvonen:** formal analysis, investigation, methodology, validation, writing – original draft preparation.

## Ethics Statement

The signed statement given by the Committee on Research Ethics of the University of Eastern Finland (Description No 5/2025) clarifies that based on the ethical review system for research in Finland, the study does not require ethical review in Finland. The statement includes the description of the ethical review system for research in Finland, and underlines that to ensure high ethical standards, research with human participants must comply with the guidelines of the Finnish National Board on Research Integrity TENK. The study participants provided their verbal informed consent to participate in the study prior to their participation, including consent to participate and consent to publication. The informed consents were given for the author of the article who conducted interviews over Zoom. The informed consents are stored on Zoom recording and interview transcripts.

## Conflicts of Interest

The authors declare no conflicts of interest.

## Data Availability

The data supporting the findings of this study are not publicly available due to privacy restrictions to protect the confidentiality of the participants.
